# Resolution of Molluscum Contagiosum After Discontinuation of Topical Corticosteroids During Dupilumab Therapy for Atopic Dermatitis: A Case Report

**DOI:** 10.7759/cureus.67391

**Published:** 2024-08-21

**Authors:** Marina Yunoki, Kensuke Fukuchi, Toshiharu Fujiyama, Taisuke Ito, Tetsuya Honda

**Affiliations:** 1 Department of Dermatology, Hamamatsu University School of Medicine, Hamamatsu, JPN

**Keywords:** topical steroid withdrawal, viral immunology, molluscum contagiosum, dupilumab, atopic dermatitis (ad)

## Abstract

A 35-year-old male patient with atopic dermatitis (AD) was referred to our department for exacerbated AD lesions. His sudden discontinuation of topical corticosteroid had induced erythroderma on his face, extremities, and trunk. Additionally, he presented small multiple whitish papules, mainly on the trunk and thighs, diagnosed as molluscum contagiosum (MC). Dupilumab was initiated in combination with a topical corticosteroid (0.05% betamethasone butyrate propionate). After four weeks, the AD symptoms substantially improved, while MC showed no changes. After 11 weeks of dupilumab therapy, he abruptly stopped topical corticosteroid treatment, and the MC lesions completely resolved in two weeks.

## Introduction

Dupilumab is a fully human monoclonal immunoglobulin G4 antibody directed against interleukin (IL)-4 receptor subunit alpha and inhibits IL-4 and IL-13 signaling. Since dupilumab exerts significant therapeutic efficacy in patients with moderate-to-severe atopic dermatitis (AD), it is now widely used to treat AD refractory to conventional topical corticosteroids and other immunosuppressive agents [1].

Molluscum contagiosum (MC) is a common cutaneous infection caused by the MC virus. MC frequently develops in children, but it occasionally occurs in immunosuppressed adults or patients with AD [2,3]. In children, MC may disappear spontaneously or with treatments by curettage or cryotherapy. In adults with AD, MC refractory to treatment may proliferate, possibly because of impaired skin barrier functions and reduced anti-viral immunity following continuous topical corticosteroid therapy [2-4].

Recently, cases of refractory MC in AD patients have spontaneously resolved during dupilumab treatment [4-7]. Here, we report a case of widespread MC in an AD patient that persisted after dupilumab therapy but, as expected, rapidly resolved following discontinuation of topical corticosteroids.

## Case presentation

**Figure 1:**
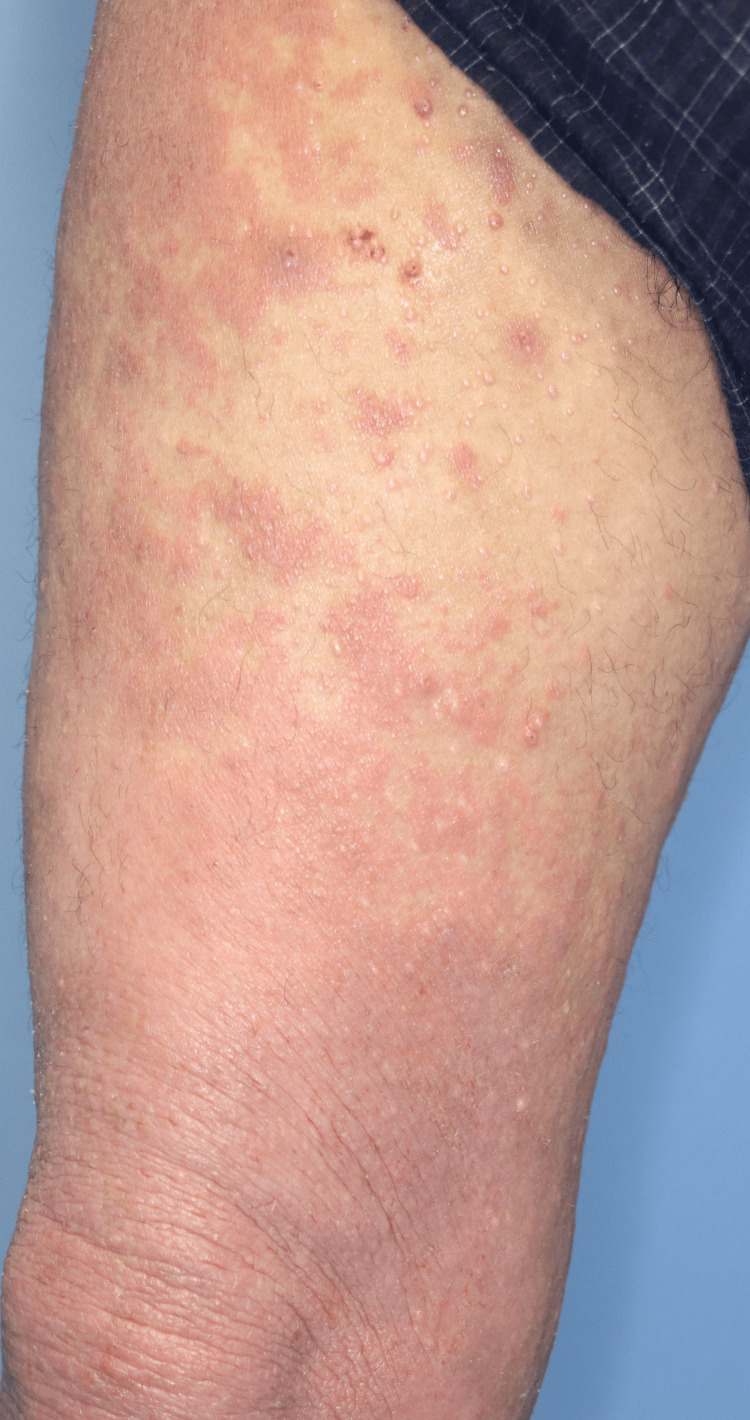
Clinical findings of molluscum contagiosum before treatment Multiple umbilicated papules (molluscum contagiosum lesions) upon an erythematous base (atopic dermatitis lesions) in the right thigh.

**Figure 2:**
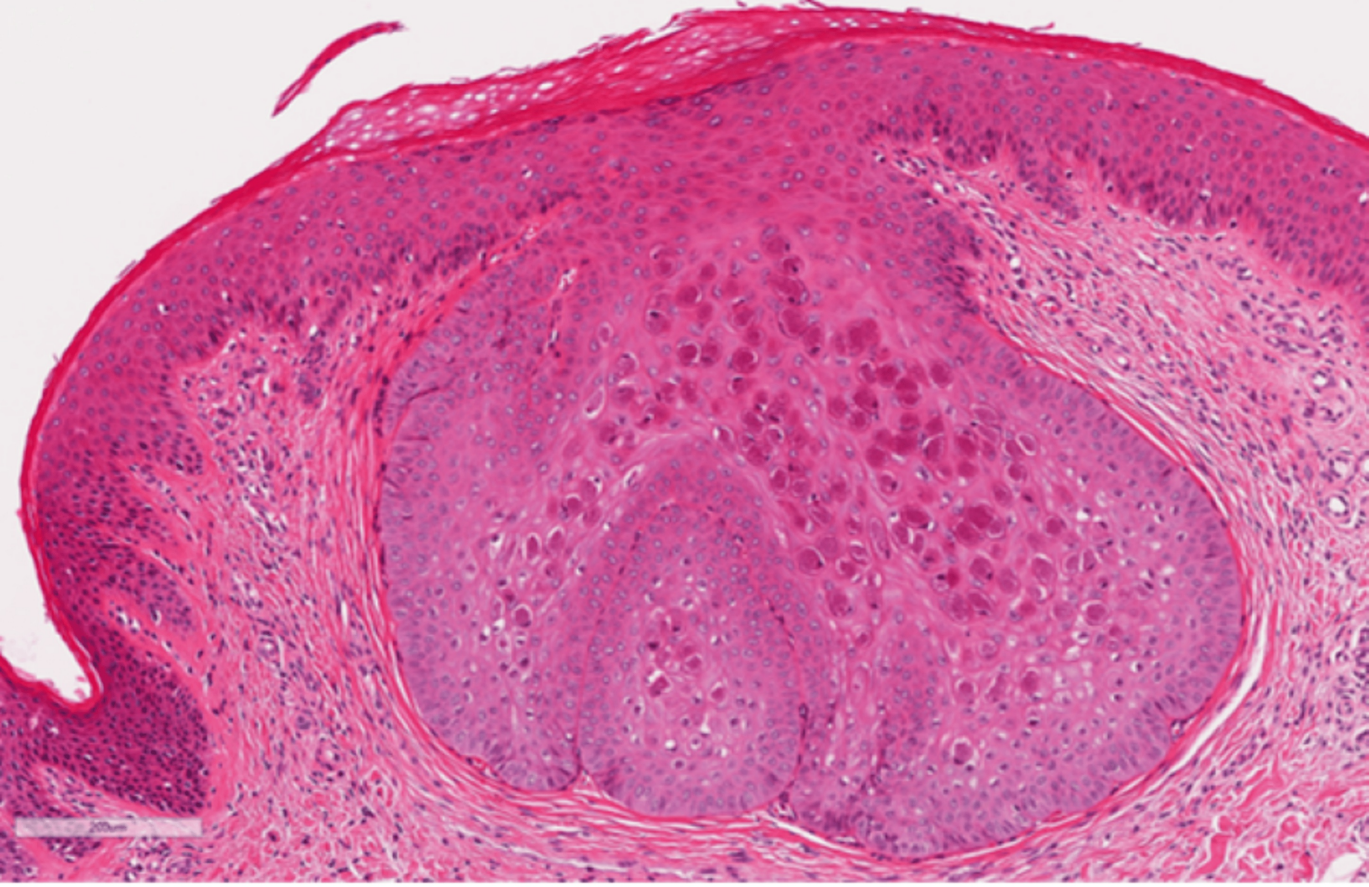
Histological findings of molluscum contagiosum Multiple large, eosinophilic “molluscum bodies” can be observed (hematoxylin-eosin stain, original magnification ×100, scale bar = 200 µm).

A 35-year-old man with AD was referred to our department for treatment of exacerbated AD lesions. Despite treatment with numerous topical corticosteroids alone over several years, the skin lesions persisted. He declined further treatment, and his AD symptoms worsened. He presented with erythroderma on his face, extremities, and trunk (Investigator Global Assessment (IGA) score of 4, Eczema Area and Severity Index (EASI) score of 63.8, body surface area (BSA) of 90%), along with small multiple whitish papules, mainly on the trunk and thighs (Figure 1). The papules were clinically diagnosed as MC, with the diagnosis supported by histological findings (Figure 2). The patient had no history of immunosuppressive drug use or immunodeficiency. An initial dose of 600 mg of dupilumab, followed by 300 mg for subsequent doses, was administered every two weeks in combination with a topical corticosteroid (0.05% betamethasone butyrate propionate).

**Figure 3:**
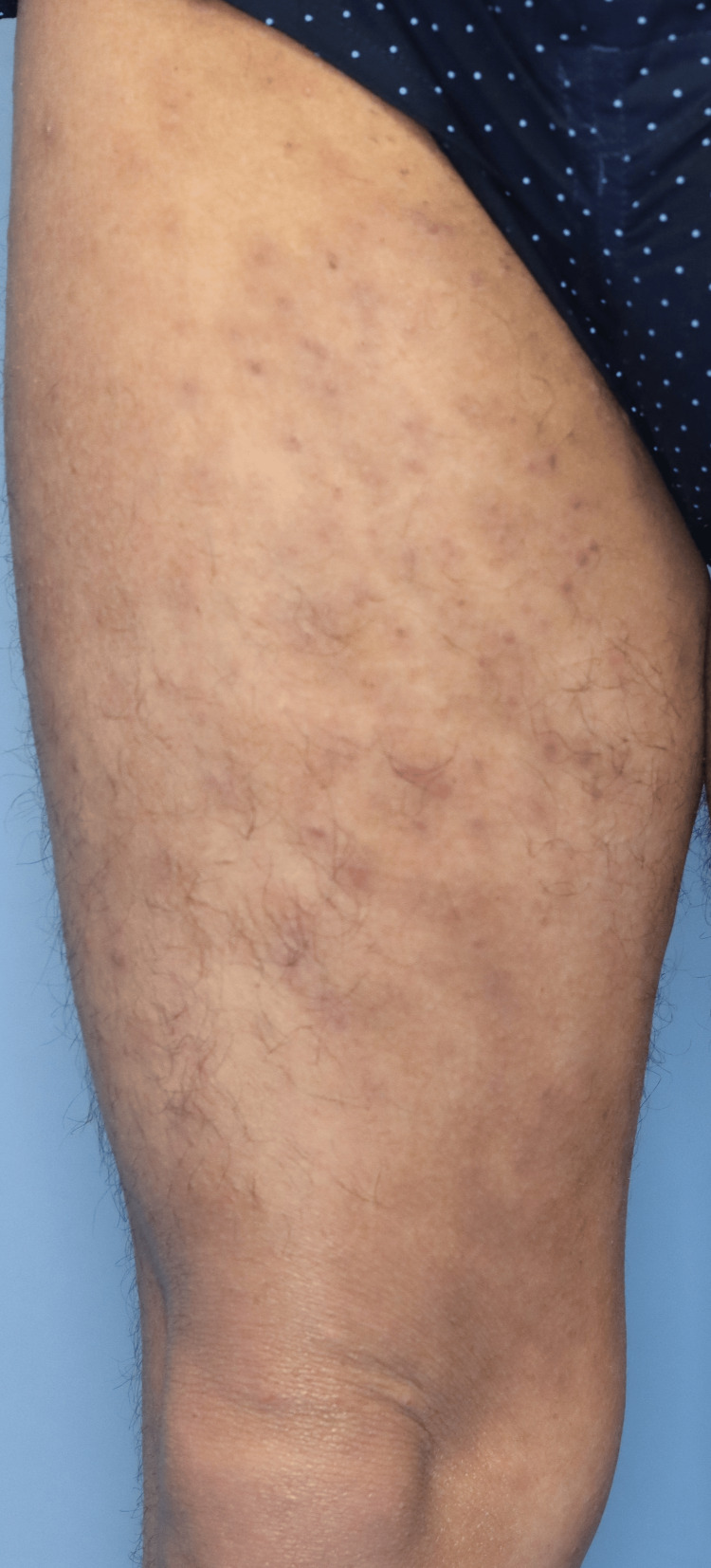
Clinical findings of molluscum contagiosum after treatment Disappearance of multiple molluscum contagiosum lesions four weeks after discontinuation of topical steroids during dupilumab treatment.

After four weeks, the AD symptoms substantially improved (IGA score, 2; EASI score, 12.0; BSA, 35%), while MC showed no changes despite cryotherapy. Curettage was also attempted but was difficult to perform due to pain, even with the use of local anesthetic cream. After 11 weeks of dupilumab therapy, he abruptly stopped topical corticosteroid treatment, although he continued with dupilumab routinely. As a result, the MC lesions began to disappear, completely resolving in two weeks (Figure 3). Some erythema persisted after 13 weeks of dupilumab therapy (IGA score, 2; EASI score, 20.6; BSA, 35%), and he returned to topical corticosteroid therapy. Thereafter, signs of MC did not recur.

## Discussion

To date, six cases of MC resolution during dupilumab treatment have been reported [4-7]. These cases involved adults (21-47 years old) with moderate to severe AD (EASI score, 16.9-46) since childhood. The duration of MC ranged from two months to 10 years, and all patients developed MC during their AD treatment. MC lesions resolved within eight to 32 weeks after initiating dupilumab. This suggests that dupilumab may enhance T helper 1 (Th1)/cytotoxic T cell type 1 cutaneous immune responses by inhibiting IL-4/IL-13 signaling, thereby improving antiviral immunity against MCV [4]. Our patient experienced MC resolution after 13 weeks of dupilumab treatment, consistent with previously reported duration.

Conversely, there have been reports of MC worsening after eight weeks of dupilumab administration and subsequent discontinuation [8]. However, this worsening was considered a transient exacerbation that could potentially have been resolved with continued administration [5,6].

In our case, cryotherapy was attempted but did not improve symptoms. The patient then abruptly stopped only the topical corticosteroid treatment after 11 weeks of initiating dupilumab therapy, believing that this might improve his residual MC lesions. Consequently, MC was cured 13 weeks after initiating dupilumab therapy. Based on this, we hypothesize that discontinuing topical corticosteroids could be a viable treatment option for MC in AD patients undergoing dupilumab therapy.

Analysis from seven randomized placebo-controlled trials indicated that combining dupilumab with topical steroids did not increase the risk of infection compared to dupilumab alone but rather resulted in a lower incidence [9]. Therefore, the combination of dupilumab and topical steroids is recommended at the start of treatment in AD patients with MC, but discontinuation of topical steroids may be considered if MC remains despite improvement of AD symptoms. Further research is warranted to explore this hypothesis and its underlying mechanisms.

## Conclusions

In conclusion, we present a case of refractory MC in a patient with AD that was successfully treated with dupilumab plus interruption of topical corticosteroids. Several reports indicate that dupilumab treatment cured MC complicated by AD. While transient inflammation and dissemination can occur, continued dupilumab treatment is expected to lead to healing. A combination of dupilumab with topical corticosteroids is advisable in AD complicated by MC, though discontinuation of topical corticosteroids may be considered once inflammation improves with dupilumab.
